# Nomograms for the Prediction of Survival for Patients with Pediatric Adrenal Cancer after Surgery

**DOI:** 10.7150/jca.36861

**Published:** 2020-02-03

**Authors:** Junjiong Zheng, Jinhua Cai, Xiayao Diao, Jianqiu Kong, Xiong Chen, Hao Yu, Weibin Xie, Jian Huang, Tianxin Lin

**Affiliations:** 1 Department of Urology, Sun Yat-Sen Memorial Hospital, Sun Yat-Sen University, Guangzhou, People's Republic of China; Guangdong Provincial Key Laboratory of Malignant Tumor Epigenetics and Gene Regulation, Sun Yat-Sen Memorial Hospital, Sun Yat-Sen University, Guangzhou, People's Republic of China; 2 Department of Neurology, Sun Yat-Sen Memorial Hospital, Sun Yat-Sen University, Guangzhou, People's Republic of China; 3 State Key Laboratory of Oncology in South China

**Keywords:** adrenal cancer, pediatric, survival, nomogram

## Abstract

**Purpose**: To develop and validate a nomogram to postoperatively evaluate overall survival (OS) and cancer-specific survival (CSS) in patients with pediatric adrenal cancer.

**Methods**: In total, 847 eligible patients diagnosed between 1988 and 2015 form the Surveillance Epidemiology, and End Results (SEER) database were enrolled in this study according to the specified inclusion and exclusion criteria. They were divided into a training set (n = 661) and a validation set (n = 186). Multivariate Cox proportional hazards regression algorithm was used to identify the independent predictors of OS and CSS in the training set, and develop the predicting models, which were presented two nomograms. The performance of the nomograms (discrimination, calibration and clinical usefulness) was assessed in the training set and validated in the validation set.

**Results**: Based on the multivariate Cox proportional hazards regression analyses, three independent predictors including age at diagnosis, tumor size and M stage were identified for both OS and CSS. Then, an OS nomogram and a CSS nomogram were developed incorporating these three predictors, respectively. The OS nomogram showed good calibration and discrimination in the training set (C-index [95% CI], 0.744 [0.711-0.777]), which was confirmed in the validation set (C-index [95% CI], 0.746 [0.656-0.836]). Favorable calibration and discrimination of the CSS nomogram were also observed in the training set (C-index [95% CI], 0.749 [0.715-0.783]) and validation set (C-index [95% CI], 0.789 [0.710-0.868]). Moreover, the nomograms successfully distinguished patients with high risk of all-cause and cancer-specific mortality in all patients and in the stratified analyses. Decision curve analysis demonstrated the usefulness of the nomograms.

**Conclusion**: The presented nomograms show favorable predictive accuracy for OS and CSS in patients with pediatric adrenal cancer after surgery. Further validation is warranted prior to clinical implementation.

## Introduction

Adrenal cancers are mainly represented by adrenocortical cancer (ACC), neuroblastoma (NB), ganglioneuroblastoma (GNB) and malignant adrenal pheochromocytoma (PCC). Apart from NB, adrenal cancer is rare in the pediatric population 1, 2. NB is childhood cancer rising from neural crest progenitor cell, accounting for nearly 10% of all childhood cancers 3-5. The most common site of origin of NB is the adrenal medulla, accounting for 35% of cases 6, and NB with adrenal site is associated with inferior survival 7.

Adrenal cancer is usually aggressive with a poor prognosis, since it has often invaded nearby tissues or metastasized to distant organs at the time of diagnosis 3, 8, 9. Complete surgical resection of the tumor is the most important and mainstay treatment for patients with adrenal cancer, which carries the best hope for prolonged survival and potential cure 10-12. However, clinical outcome varies even in homogenously treated adrenal cancer patients with the same tumor stage because of the heterogeneous nature of adrenal cancer 9, 13, 14. Indeed, if clinicians can identify patients at high risk after surgery, then systemic therapy can be implemented in time. Therefore, it's of great significance to construct a prognostic evaluation tool to aid in clinical decision making, facilitating the personalized and precision management of patients with adrenal cancer.

Nowadays, knowledge has grown regarding that the clinical manifestations and biologic behavior of pediatric adrenal cancer is different from that in adult adrenal cancer 2, 15-17. For example, NB is unique to the pediatric age group and does not have adult counterparts 3, 4. Pediatric patients with ACC seem to present more endocrine dysfunction features and *TP53* mutations than adult patients 16. Hypertension may be continuous rather than paroxysmal in pediatric PCC 2. And the genomic characteristics of PCC are also different between children and adults 18, 19. Therefore, the prognosis predictors of adrenal cancer are different between pediatric and adult patients. However, to our knowledge, a prognostic prediction tool has not been proposed specifically for pediatric adrenal cancer patients.

Hence, this study aimed to develop and validate nomograms for the postoperative survival prediction in individual patients with pediatric adrenal cancer using the Surveillance Epidemiology, and End Results (SEER) database.

## Methods and Materials

### Patients

In total, 847 adrenal cancer patients diagnosed between 1988 and 2015 from the SEER database were enrolled in this study according to the specified inclusion and exclusion criteria. Inclusion criteria consisted of the following: (a) adrenal cancer patients confirmed by pathology; (b) underwent surgery of primary site; (c) age at diagnosis less than 20; and (d) clinicopathological data and follow-up information available. Exclusion criteria included the following: (a) patients suffered from other cancer disease; (b) patients with bilateral adrenal cancer. The pathway of patient selection is shown in Supplementary Figure S1. All enrolled patients were divided into two cohorts: 661 patients diagnosed between 2005 and 2015 were allocated to the training set, while 186 patients diagnosed between 1988 and 2004 were allocated to the validation set.

Clinicopathological data extracted for each case included age at diagnosis, sex, tumor laterality, tumor size, tumor invasion, N stage and M stage. Follow-up data extracted for each case included survival status, survival time, and cause of death. Overall survival (OS) duration was defined as time from diagnosis until death or last follow-up. Cancer-specific survival (CSS) duration was defined as time from diagnosis until death because of adrenal cancer or last follow-up.

### Nomograms Construction and Performance Assessment

Clinicopathological candidate predictors, including histological type, were tested using a multivariate Cox proportional hazards regression algorithm in the training set. Backward stepwise selection using Akaike's Information Criterion (AIC) was applied to select the significant predictors of OS and CSS 20. Then, an OS nomogram and a CSS nomogram were constructed based on the multivariate Cox proportional hazards regression models, respectively.

The performance of the nomograms was evaluated with respect to their discrimination and calibration in the training set. The Harrell's C-index was applied to quantitatively evaluate the discriminative ability, which is commonly used to assess the discrimination of prognostic models 21. Note that bootstrapping using 1000 resampling procedures was used to obtain the C-index that was corrected for potential overfitting. The calibration of the nomograms was evaluated by plotting the calibration curves, which compared the nomogram-predicted survival probability with the observed survival probability.

### Validation of the Nomograms

The performance of the two nomograms was validated in the validation set, respectively. The multivariate Cox proportional hazards regression formulas constructed using the training set were applied to all patients of the validation set, with risk scores calculated for each patient to reflect the risk of all-cause and cancer-specific mortality. Cox proportional hazards regression was then performed by using the risk score as a factor in the validation set. Finally, based on the regression analyses, the C-indices were calculated and the calibration curves were plotted to validate the performance of the nomograms.

### Categorization of Patients into High- or Low-risk Groups

A risk score for each patient was calculated based on the multivariate Cox proportional hazards regression formula. Then all patients were divided into high-risk and low-risk groups based on the optimal risk score cutoff value, which was identified by using X-tile plots in the training set 22. The difference in the survival curves of the high-risk and low-risk groups was assessed by using the log-rank test. Moreover, stratified analyses were also performed within various subgroups in the combined training and validation set.

### Clinical Usefulness of the Nomograms

The decision curve analysis (DCA) was used to estimate the clinical usefulness of the proposed nomograms by calculating the net benefits at different threshold probabilities. The DCA algorithm can serves as a comprehensive method for assessing and comparing different diagnostic and prognostic models 23.

### Incremental predictive value of histologic grade

Since histologic grade has been reported as a factor associated with prognostic in ACC and NB patients, we performed additional analyses to explore whether it adds to the value of the presented nomograms 24, 25. In all 847 patients, only 491 patients recorded the information about the histologic grade. Therefore, the incremental value of histologic grade as an additional candidate predictor was assessed in this dataset, with C-indices calculated, calibration curves plotted and DCA performed.

### Statistical Analyses

The X-tile software version 3.6.1 (Yale University School of Medicine, New Haven, CT, USA) was used to create the X-tile plots. X-tile plots provide a single method to automatically select the optimum cutoff based on the highest χ² value (i.e., minimum *P* value) defined using a Kaplan-Meier survival analysis and the log-rank test 22. All other statistical analyses were performed using R statistical software version 3.5.1 (https://www.r-project.org/). The “survival” package and “MASS” package were used to perform the Cox proportional hazards regression model analysis. The nomograms and calibration plots were produced using the “rms” package. DCA was performed using the function “stdca.R.” All statistical tests were two-tailed, and *P* < 0.05 were deemed significant.

## Results

### Patient Clinicopathological Characteristics

The clinicopathological characteristics of the patients in the training and validation sets are presented in Table 1 and Supplementary Table S1. In total, 77.2% and 13.0% of patients were diagnosed as NB and GNB, respectively. And ACC accounted for 7.6% of all patients (Supplementary Figure S2). As for the distribution of age at diagnosis, more than 73% patients were diagnosed no more than 3 years of age. A single peak was seen in 1 years of age, and similar findings were also found in male and female subgroups (Supplementary Figure S3A). However, the distribution characteristics of age at diagnosis vary from different tumor types (Supplementary Figure S3B). Among all enrolled patients, 193 patients (22.8%) were dead during the follow-up, and 174 patients (20.5%) died due to adrenal cancer. Median fellow-up was 4.3 years (Interquartile range, 1.8-8.5). There was no significant difference between the OS (*P* = 0.310) or CSS (*P* = 0.260) of patients with different histological types (Supplementary Figure S4).

### Nomograms Construction and Performance Assessment

**Figure 1:**
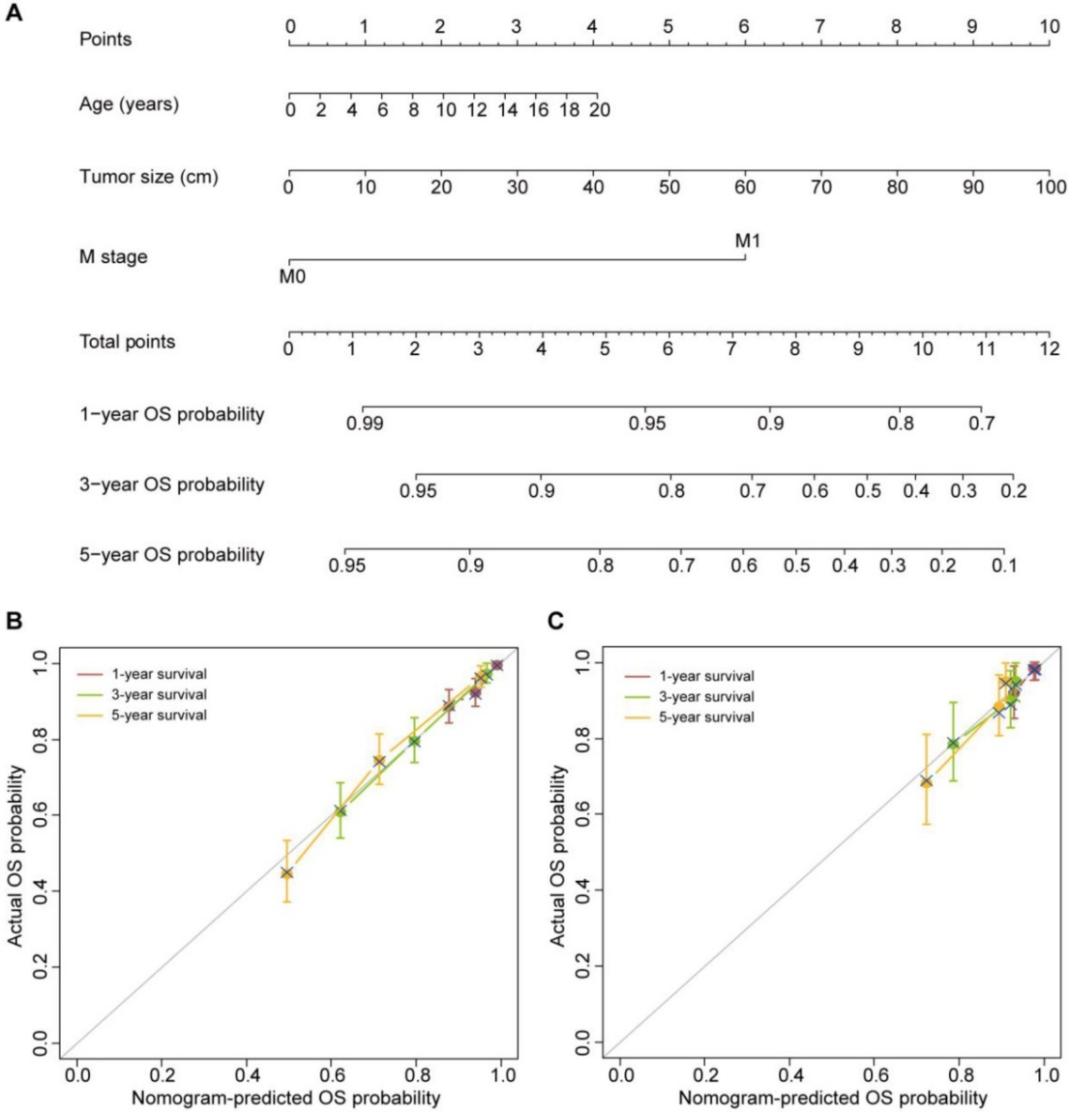
**The OS nomogram and its performance. (A)** The OS nomogram developed to estimate the OS probability for adrenal cancer patients after surgery. **(B)** Calibration curves of the OS nomogram in the training set. **(C)** Calibration curves of the OS nomogram in the validation set. The calibration curves depict the calibration of the nomogram in terms of the agreement between the predicted and observed 1-, 3- and 5-year OS probability. The 45-degree gray line represents perfect calibration. The broken line represents the predictive performance of the nomogram: a closer fit to the ideal line indicates a better prediction.

Age at diagnosis, tumor size and M stage were identified as independent predictors of OS based on the multivariate Cox proportional hazards regression algorithm (Table 2). Then, the OS nomogram was constructed by incorporating these three predictors based on the multivariate Cox proportional hazards regression model (Figure 1A). The OS nomogram showed favorable discrimination with a C-index of 0.744 (95% CI, 0.711-0.777) in the training set. The calibration curves for the 1-, 3- and 5-year OS showed favorable agreement between the nomogram-predicted OS probability and actual OS probability, indicating good calibration of the OS nomogram in the training set (Figure 1B).

**Figure 2:**
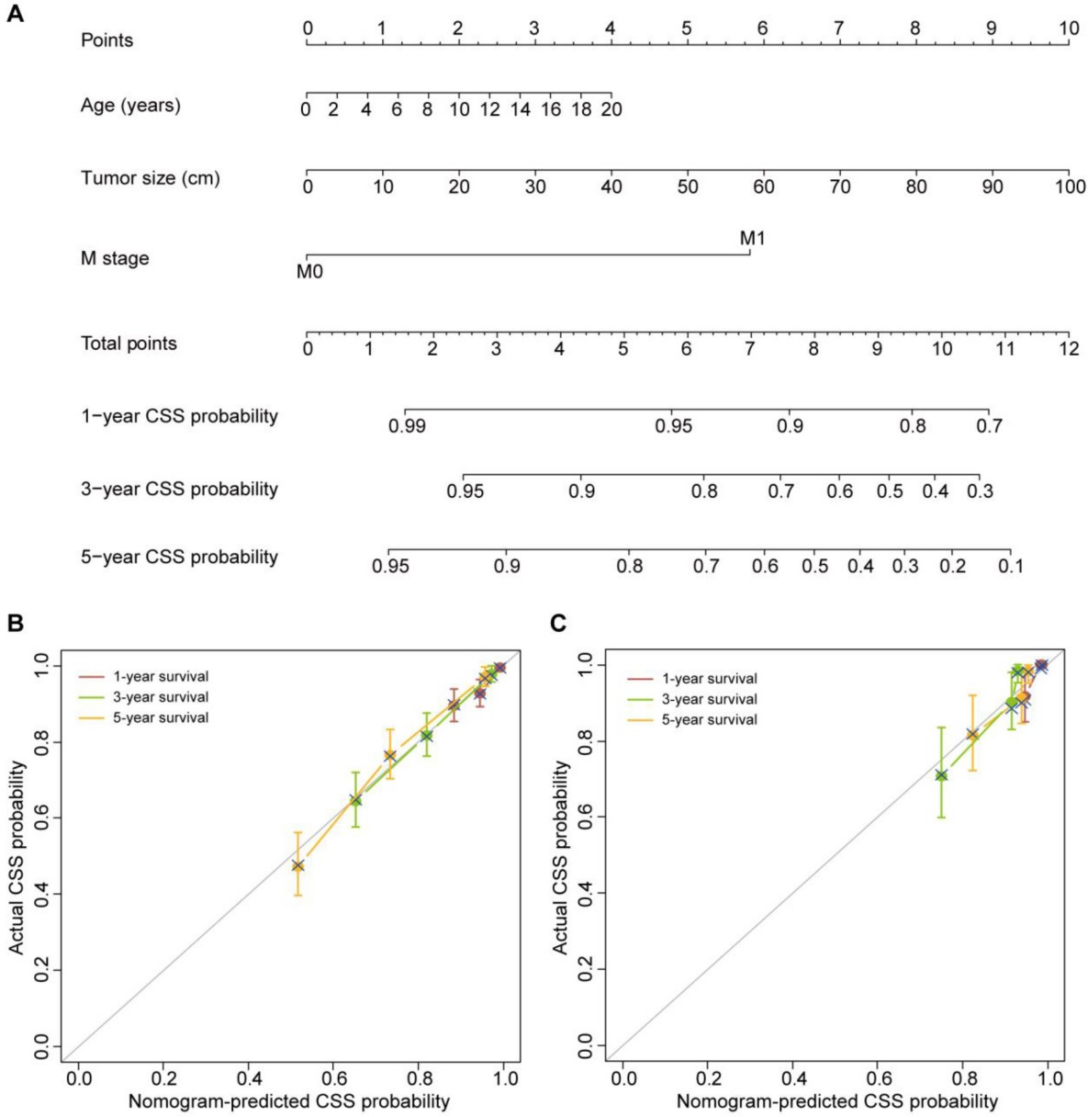
** The CSS nomogram and its performance. (A)** The CSS nomogram developed to estimate the CSS probability for adrenal cancer patients after surgery. **(B)** Calibration curves of the CSS nomogram in the training set. **(C)** Calibration curves of the CSS nomogram in the validation set. The calibration curves depict the calibration of the nomogram in terms of the agreement between the predicted and observed 1-, 3- and 5-year CSS probability. The 45-degree gray line represents perfect calibration. The broken line represents the predictive performance of the nomogram, which has a closer fit to the ideal line indicating a better prediction.

The three variables, including age at diagnosis, tumor size and M stage, were also found to be independent predictors of CSS based on the multivariate Cox proportional hazards regression algorithm (Table 3). The CSS nomogram was developed by incorporating these predictors (Figure 2A). The CSS nomogram yielded a C-index of 0.749 (95% CI, 0.715-0.783). The calibration curves for the 1-, 3- and 5-year CSS also showed favorable calibration of the CSS nomogram in the training set (Figure 2B).

### Validation of the Nomograms

The favorable discrimination of the OS nomogram was confirmed using the validation set (C-index [95% CI], 0.746 [0.656-0.836]). And good calibration of the OS nomogram was also observed in the validation set (Figure 1C). As for the CSS nomogram, the C-index was 0.789 (95% CI, 0.710-0.868). The calibration curves for the 1-, 3- and 5-year CSS in the validation set also confirmed the good calibration of the CSS nomogram (Figure 2C).

### Categorization of Patients into High- or Low-risk Groups

The risk score was calculated for OS and CSS by using the following formulas:

OS risk score = 0.074 × age at diagnosis + 0.037 × tumor size + 2.192 × M (with distant metastasis).

CSS risk score = 0.078 × age at diagnosis + 0.039 × tumor size + 2.259 × M (with distant metastasis).

Note that the indicator function (M) is equal to 1 if the statement in the parentheses is true and is equal to 0 otherwise.

**Figure 3:**
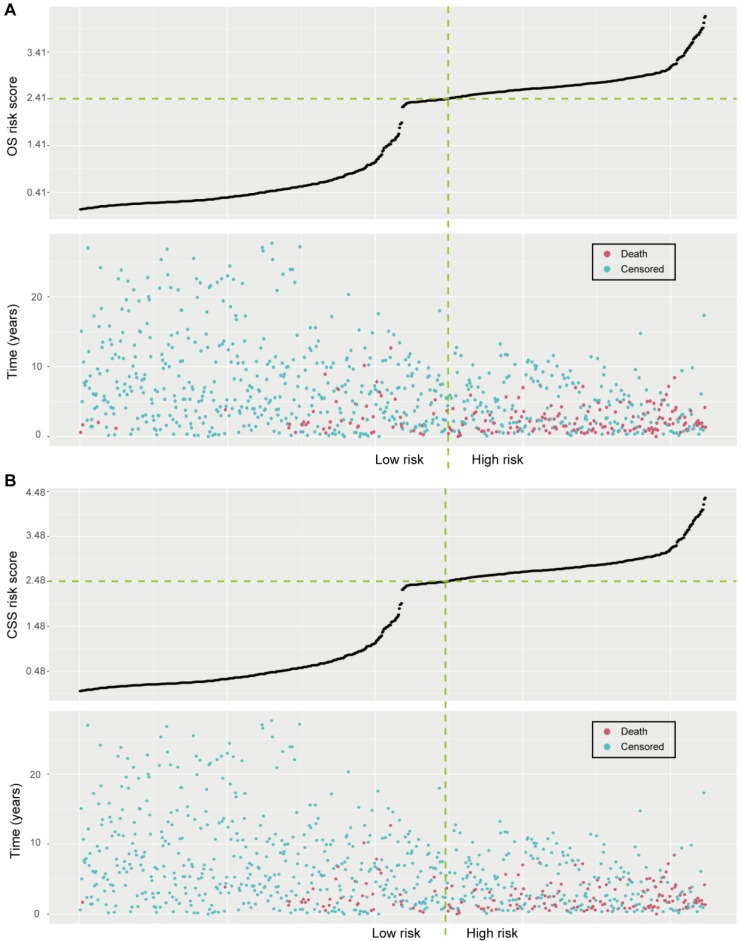
** Risk score analyses in the combined training and validation set. (A)** Distributions of the OS risk score and OS status of individual patients. **(B)** Distributions of the CSS risk score and CSS status of individual patients.

**Figure 4:**
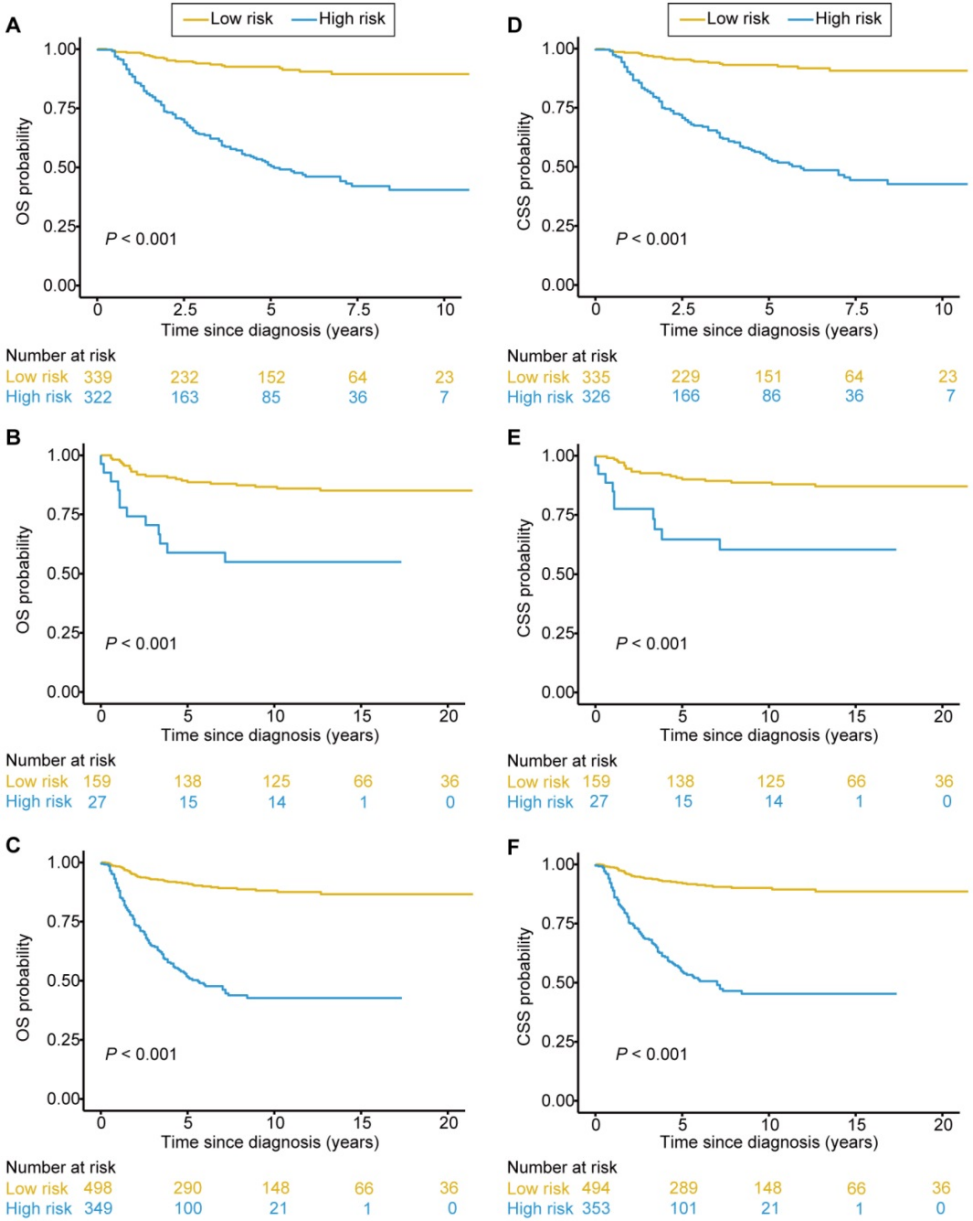
** Kaplan-Meier survival curves categorized into low-risk and high-risk groups.** Significant discrimination between the OS of the high-risk and low-risk patients was observed in the training set **(A)**, the validation set **(B)**, and the combined training and validation set **(C)**. Significant discrimination between the CSS of the high-risk and low-risk patients was also observed in the training set **(D)**, the validation set **(E)**, and the combined training and validation set **(F)**.

The optimal OS risk score cutoff generated by the X-tile plots was 2.41 (Supplementary Figure S5A-C). All patients were classified into high-risk and low-risk groups according to the optimal cutoff value. We assessed the distributions of the OS risk score and OS status in the combined training and validation set, and found that patients with higher risk scores were more likely to have death (Figure 3A). There was a significant discrimination between the OS of the high-risk and low-risk patients in the training set (Figure 4A), which was confirmed in the validation set (Figure 4B). The OS risk score was also associated with the OS in the combined training and validation set (*P* < 0.001, Figure 4C) and in the stratified analyses (Supplementary Figure S6). Thus, the OS nomogram can successfully distinguish patients with high risk of all-cause mortality.

As for the CSS risk score, we defined an optimal cutoff value of 2.48 based on the X-tile plots (Supplementary Figure S5D-F). Accordingly, the patients were categorized into high-risk and low-risk groups for CSS. The distributions of the CSS risk score and CSS status in all patients are shown in Figure 3B. Patients with higher risk scores were more likely to have death due to adrenal cancer. Significant discrimination between the CSS of the high-risk and low-risk patients was observed both in the training and validation sets (Figure 4D and 4E, respectively). The CSS risk score was also associated with the CSS in the combined training and validation set (*P*<0.001, Figure 4F) and in the stratified analyses (Supplementary Figure S7). Therefore, those patients with high risk of cancer-specific mortality can be identified by using our CSS nomogram.

### Clinical Usefulness of the Nomograms

**Figure 5:**
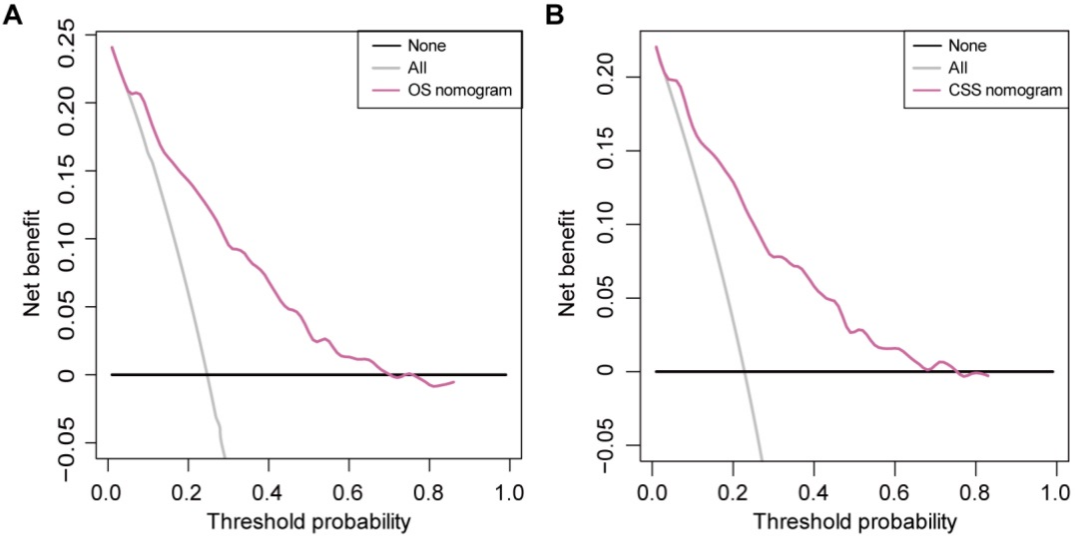
** DCA for the nomograms. (A)** DCA for the OS nomogram. **(B)** DCA for the CSS nomogram. The net benefit was plotted versus the threshold probability. The net benefit was calculated by subtracting the proportion of all patients who are false positive from the proportion who are true positive, weighting by the relative harm of forgoing treatment compared with the negative consequences of an unnecessary treatment. The gray and black lines depict the net benefit of the strategy of treating all patients and no patients, respectively. The red line represents the nomogram.

The DCAs of the OS nomogram and CSS nomogram are presented in Figure 5. The OS nomogram and the CSS nomogram offered a net benefit over the ''treat-all'' or “treat-none” strategy at a threshold probability < 71% and < 76% at 5 years, respectively. In addition, similar DCA findings were also observed in both the training and validation sets (Supplementary Figure S8). Therefore, the presented nomograms are clinically useful.

### Incremental predictive value of histologic grade

The new models after the addition of histologic grade are presented in Supplementary Figure S9A and S10A (defined as OS nomogram II and CSS nomogram II, respectively). The calibration curves demonstrated good calibration for the OS nomogram II and CSS nomogram II (Supplementary Figure S9B and S10B, respectively). However, C-indices indicated that incorporating histologic grade did not improve prediction performance for either the OS nomogram II (C-index [95% CI], 0.765 [0.722-0.808] *vs*. 0.766 [0.723-0.809]) or the CSS nomogram II (C-index [95% CI], 0.775 [0.733-0.817] *vs*. 0.774 [0.732-0.816]). The DCA also indicated that the paired nomograms had similar performance, with comparable net benefit at different threshold probabilities (Supplementary Figure S9C and S10C).

## Discussion

Our study demonstrates that age at diagnosis, tumor size and M stage were independent predictors of OS or CSS in patients with pediatric adrenal cancer after surgery. To provide easy-to-use tools for the postoperative survival prediction in individual patients, an OS nomogram and a CSS nomogram were developed incorporating these predictors, respectively. The nomograms showed good discrimination and calibration in the training and validation set, which may aid in clinical decision-making.

Over the past several decades, the outcome for childhood cancer has dramatically improved. However, the long-term outcome of pediatric adrenal cancer patients with high risk remains poor 9, 17, 26. Pediatric adrenal cancer is a heterogeneous malignant neoplasm with prognosis ranging from near uniform survival to high risk for fatal demise. The heterogeneity of the outcome makes it difficult to provide an assessment of the prognosis after surgery of the pediatric adrenal cancer 9, 13, 14. Indeed, if clinicians can identify patients at high risk after surgery, then systemic therapy can be implemented in time. Therefore, accurately estimating the prognosis can optimize disease management and may improve patient outcome.

As far as we know, research that specifically focus on risk factors for outcomes of pediatric NB located at adrenal has not been reported 27. In addition, maybe due to the low incidence of ACC, only a few prognostic prediction models have been reported for ACC patients previously 24, 28, 29. However, further studies are warranted due to limitations. For example, cases with insufficient data were not excluded for analysis, and “unknown” was treated as a category in some variables 24, 28; age at diagnosis was used as a categorical variable rather than continuous variable 24; a useless variable “year of diagnosis” was even identified as an independent risk factor for survival and incorporated in the final model 24. More importantly, these models were developed in ACC patients of all ages, which neglected that the prognosis predictors of ACC are different between pediatric and adult patients 24, 28, 29. Due to the deficient number of cases, it is hard to develop reliable models for the rare tumors, like the pediatric ACC. In view of the above, the proposed nomograms were developed based on the pediatric adrenal cancer patients in this study, including various histological types. Note that histological type was used as a candidate predictor for regression analyses in our study. However, it was not associated with OS or CSS among pediatric adrenal tumor patients based on the regression analyses. Then stratified analyses were performed in the histological type subgroups to confirm whether the models were applicable to different tumor types. Encouragingly, our nomograms performed well in different histological type subgroups as well. Therefore, although some prognostic prediction models for adrenal cancer have been reported as mentioned above, our study did do a lot of improvement compared with previous studies.

In this study, age at diagnosis, tumor size and M stage were identified as independent predictors of OS or CSS in patients with pediatric adrenal cancer after surgery. Older age at diagnosis was an adverse prognostic factor in our study, which was consistent with the results reported in the previous studies on ACC or pediatric NB 27, 29. Tumor size also played an important role in predicting prognosis of pediatric patients with adrenal cancer after surgery. In our study, tumor size was used as a continuous variable rather than categorical variable, which could provide more detailed information, thus improving the model performance. For many different types of pediatric adrenal caner, the staging is based on information and data primarily from adult populations. Tumor size with 5 cm is often used as a cutoff for grouping patients in terms of tumor stage for some types of adrenal cancer. However, it is reasonable to suspect that this tumor size cutoff may be inappropriate to pediatric patients due to the different body size and different characteristics of adrenal cancer between pediatric and adult patients. We tried to explore the ideal cutoff for the tumor size using X-tile plots in all enrolled patients. As a result, an optimal cutoff value of 10.0 cm was defined, which was longer than 5 cm. This result also indicated that staging system should be established specifically for pediatric adrenal cancer patients for better disease management. M stage is another well-established prognostic variable in pediatric adrenal cancer 9, 30-32. Our study further elucidated that even after surgical, patients with distant metastasis disease had significant worse prognosis than those without.

To our knowledge, this is the first attempt to develop and validate nomograms for the survival prediction in individual patients with pediatric adrenal cancer after surgery. Our study has several strengths. First, the presented prognosis models are specifically for the pediatric patients, which can better reflect the characteristics of this population. Since the clinical manifestations and biologic behavior of pediatric adrenal cancer is different from that in adult adrenal cancer, the prognosis predictors of adrenal cancer are not the same between pediatric and adult patients. Therefore, it is greatly needed to develop prognosis models specifically for these two different populations, respectively. Second, our nomograms are applicable to different histological types of pediatric adrenal cancer, which provides user-friendly tools for clinicians and patients, especially for those with rare tumor types. Third, our study indicated that tumor size is an independent predictor of OS or CSS in patients with pediatric adrenal cancer after surgery, and the optimal cutoff for tumor size we discovered is quite different from the tumor size cutoff defined by the current staging system for some types of adrenal cancer. This reminds us that we should establish staging system specifically for pediatric adrenal cancer patients rather than using the current staging system which is derived from the data of adult populations.

Despite the strengths, some unavoidable limitations of our study should be considered. First, due to the retrospective nature of the study and the strict inclusion and exclusion criteria used, potential selection biases might occur. For instance, patients suffered from other cancer disease or with bilateral adrenal cancer were excluded in our study, which will limit the application of our models to these patients. These criteria introduced selection bias by removing patients with worse prognosis (i.e., patients suffered from other cancer disease). And the selection bias thus limits our model only accurate in specific patient population. Second, data from the SEER database also suffers from lack of detail. The selected candidate factors were based on our clinical experiences, previously published studies and the available data from the SEER database. Those unrecorded clinical characteristics might also be associated with patient outcome, such as manifestation, comorbidity, mitotic index et al. In addition, the presented nomograms do not include data on molecular markers, which may serve as promising predictors. Thus, further studies are warranted to address this issue. Third, although a validation set was used for model validation in our study, further external validation in other datasets is warranted to confirm the generalizability of our nomograms before clinical application.

In conclusion, we proposed two nomograms for the OS and CSS prediction in individual patients with pediatric adrenal cancer after surgery, respectively. The nomograms showed favorable prediction efficiency, which was validated in the validation set.

## Supplementary Material

Supplementary figures and table.

## Figures and Tables

**Table 1:** Baseline clinicopathological characteristics of the patients by the OS nomogram assessment set.

	Training set (n = 661)				Validation set (n = 186)		
	No. of patients	Low risk (%)	High risk (%)		No. of patients	Low risk (%)	High risk (%)
**Age, years**							
Median (IQR^†^)	2 (1-4)	1 (0-3)	3 (2-4)		1 (0-3)	1 (0-3)	2 (1-3)
**Sex**							
Male	366	173 (47.3%)	193 (52.7%)		100	81 (81.0%)	19 (19.0%)
Female	295	166 (56.3%)	129 (43.7%)		86	78 (90.7%)	8 (9.3%)
**Laterality**							
Left	359	181 (50.4%)	178 (49.6%)		112	94 (83.9%)	18 (16.1%)
Right	302	158 (52.3%)	144 (47.7%)		74	65 (87.8%)	9 (12.2%)
**Tumor size, cm**							
Median (IQR^†^)	6.8 (4.7-10.3)	5.1 (3.5-8.6)	8.7 (6.3-11.3)		6.5 (4.0-10.0)	6.0 (3.8-9.5)	8.0 (6.4-11.7)
**Histological type**							
Adrenocortical cancer	43	30 (69.8%)	13 (30.2%)		21	18 (85.7%)	3 (14.3%)
Ganglioneuroblastoma	80	50 (62.5%)	30 (37.5%)		30	30 (100%)	0 (0%)
Neuroblastoma	526	250 (47.5%)	276 (52.5%)		128	104 (81.3%)	24 (18.7%)
Others	12	9 (75.0%)	3 (25.0%)		7	7 (100%)	0 (0%)
**Tumor invasion**							
No extra-adrenal invasion	362	254 (70.2%)	108 (29.8%)		114	108 (94.7%)	6 (5.3%)
Local invasion	58	25 (43.1%)	33 (56.9%)		23	19 (82.6%)	4 (17.4%)
Adjacent organs invasion^‡^	241	60 (24.9%)	181 (75.1%)		49	32 (65.3%)	17 (34.7%)
**N stage**							
N0	339	234 (69.0%)	105 (31.0%)		110	103 (93.6%)	7 (6.4%)
N1	322	105 (32.6%)	217 (67.4%)		76	56 (73.7%)	20 (26.3%)
**M stage**							
M0	284	281 (98.9%)	3 (1.1%)		155	155 (100%)	0 (0%)
M1	377	58 (15.4%)	319 (84.6%)		31	4 (12.9%)	27 (87.1%)

† IQR: interquartile range. ‡ Adjacent organs include kidney, diaphragm, great vessels, pancreas, spleen, and liver. Data are n or n (%) unless otherwise indicated.

**Table 2:** Univariate and multivariate Cox regression analyses of clinicopathologic factors with overall survival in the training set.

	Univariate analyses			Multivariate analyses	
	HR (95%CI)	*P*		HR (95%CI)	*P*
**Age** (continuous)	1.067 (1.035-1.100)	< 0.001*		1.077 (1.042-1.113)	< 0.001*
**Sex** (male *vs.* female)	0.919 (0.671-1.260)	0.601		-	-
**Laterality** (left *vs.* right)	0.808 (0.589-1.108)	0.186		-	-
**Tumor size** (continuous)	1.024 (1.012-1.036)	< 0.001*		1.037 (1.020-1.054)	< 0.001*
**Histological type**					
Adrenocortical cancer	Reference			-	-
Ganglioneuroblastoma	0.863 (0.425-1.756)	0.685		-	-
Neuroblastoma	0.757 (0.418-1.369)	0.357		-	-
Others	0.225 (0.029-1.728)	0.151		-	-
**Tumor invasion**					
No extra-adrenal invasion	Reference			-	-
Local invasion	1.676 (0.936-2.999)	0.082		-	-
Adjacent organs invasion	2.339 (1.679-3.258)	< 0.001*		-	-
**N stage** (N0 *vs.* N1)	2.025 (1.465-2.799)	< 0.001*		-	-
**M stage** (M0 *vs.* M1)	7.833 (4.732-12.960)	< 0.001*		8.958 (5.308-15.115)	< 0.001*

^*^ *P* < 0.05

**Table 3:** Univariate and multivariate Cox regression analyses of clinicopathologic factors with cancer-specific survival in the training set.

	Univariate analyses			Multivariate analyses	
	HR (95%CI)	*P*		HR (95%CI)	*P*
**Age** (continuous)	1.071 (1.038-1.105)	< 0.001*		1.081 (1.045-1.118)	< 0.001*
**Sex** (male *vs.* female)	0.934 (0.672-1.298)	0.683		-	-
**Laterality** (left *vs.* right)	0.772 (0.554-1.075)	0.126		-	-
**Tumor size** (continuous)	1.025 (1.013-1.037)	< 0.001*		1.040 (1.023-1.057)	< 0.001*
**Histological type**					
Adrenocortical cancer	Reference			-	-
Ganglioneuroblastoma	0.822 (0.402-1.683)	0.592		-	-
Neuroblastoma	0.682 (0.376-1.238)	0.209		-	-
Others	0.224 (0.029-1.721)	0.150		-	-
**Tumor invasion**					
No extra-adrenal invasion	Reference			-	-
Local invasion	1.896 (1.052-3.419)	0.033*		-	-
Adjacent organs invasion	2.459 (1.734-3.487)	< 0.001*		-	-
**N stage** (N0 *vs.* N1)	2.166 (1.539-3.046)	< 0.001*		-	-
**M stage** (M0 *vs.* M1)	8.172 (4.786-13.960)	< 0.001*		9.572 (5.466-16.763)	< 0.001*

^*^ *P* < 0.05

## Abbreviations

ACC: adrenocortical cancer
NB: neuroblastoma
GNB: ganglioneuroblastoma
PCC: pheochromocytoma
SEER: Surveillance Epidemiology, and End Results
OS: overall survival
CSS: cancer-specific survival
AIC: Akaike's Information Criterion
DCA: decision curve analysis
