# Supplementary material for: Nomograms for the Prediction of Survival for Patients with Pediatric Adrenal Cancer after Surgery
Source: J Cancer. 2020 Feb 3;11(8):2080–90. doi: 10.7150/jca.36861 (PMC7052927; doi:10.7150/jca.36861)

Supplementary Material

Supplementary Table 2

Table S1. Baseline characteristics of the patients in the training and validation sets. .. 2

Supplementary Figures 3

Figure S1. The pathway of patient selection in this study. 3

Figure S2. Distribution of adrenal carcinoma patients with different histological types in all enrolled patients. 4

Figure S3. Density plots of age at diagnosis in all enrolled patients. 5

Figure S4. Kaplan-Meier survival curves of all enrolled patients categorized into different histological type groups. 6

Figure S5. X-tile plots identifying the optimal risk score cutoff based on OS and CSS. 7

Figure S6. Kaplan-Meier survival curves (OS) categorized into low-risk and high-risk groups in stratified analyses in the combined training and validation set. 8

Figure S7. Kaplan-Meier survival curves (CSS) categorized into low-risk and high-risk groups in stratified analyses in the combined training and validation set. 9

Figure S8. DCA for the nomograms performed in the training and validation sets. .. 10

Figure S9. The OS nomogram II and its performance. 11

Figure S10. The CSS nomogram II and its performance. 12

Supplementary Table

Table S1. Baseline characteristics of the patients in the training and validation sets.

	Training set (n = 661)	Validation set (n = 186)
**Age, years**		
Median (Interquartile range)	2 (1–4)	1 (0–3)
**Sex**		
Male	366	100
Female	295	86
**Laterality**		
Left	359	112
Right	302	74
**Tumor size, cm**		
Median (Interquartile range)	6.8 (4.7–10.3)	6.5 (4.0–10.0)
**Tumor invasion**		
No extra-adrenal invasion	362	114
Local invasion	58	23
Adjacent organs invasion*	241	49
**N stage**		
N0	339	110
N1	322	76
**M stage**		
M0	284	155
M1	377	31
**Histologic grade†**		
Grade I	7	13
Grade II	6	7
Grade III	312	49
Grade IV	68	29
Unknown	268	88
**Survival status**		
Alive	503	151
Dead	158	35
Dead (due to adrenal cancer)	145	29

* Adjacent organs include kidney, diaphragm, great vessels, pancreas, spleen, and liver.

† Grade I = well differentiated, Grade II = moderately differentiated, Grade III = poorly differentiated, and Grade IV = undifferentiated.

Supplementary Figures

Figure S1. The pathway of patient selection in this study.

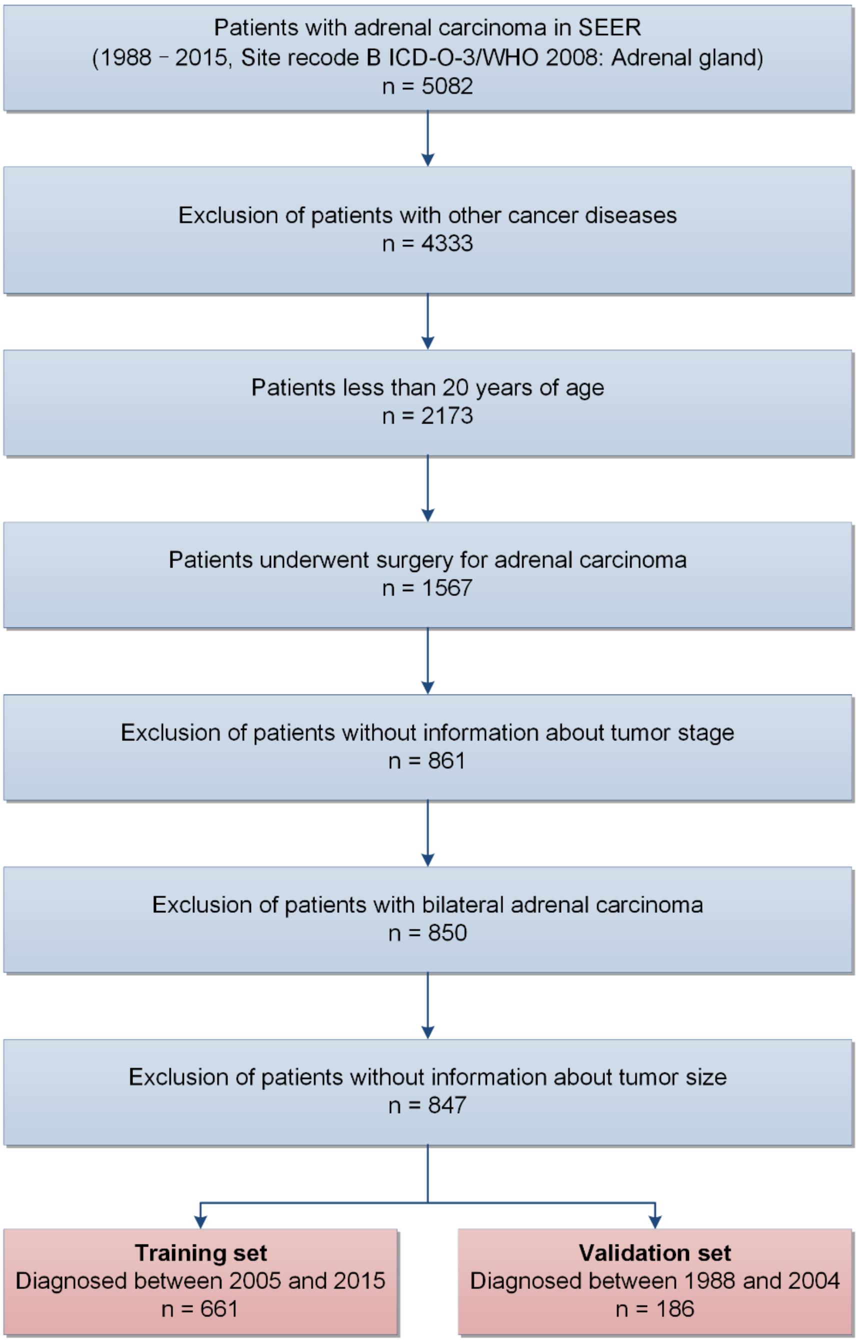

Figure S2. Distribution of adrenal carcinoma patients with different histological types in all enrolled patients.

There were only 19 patients in the *Others* group, including 13 pheochromocytoma, 1 desmoplastic small round cell tumor, 1 nephroblastoma, 1 Yolk sac tumor, 1 primitive neuroectodermal tumor, 1 neuroepithelioma, and 1 neoplasm with no specific histological type.

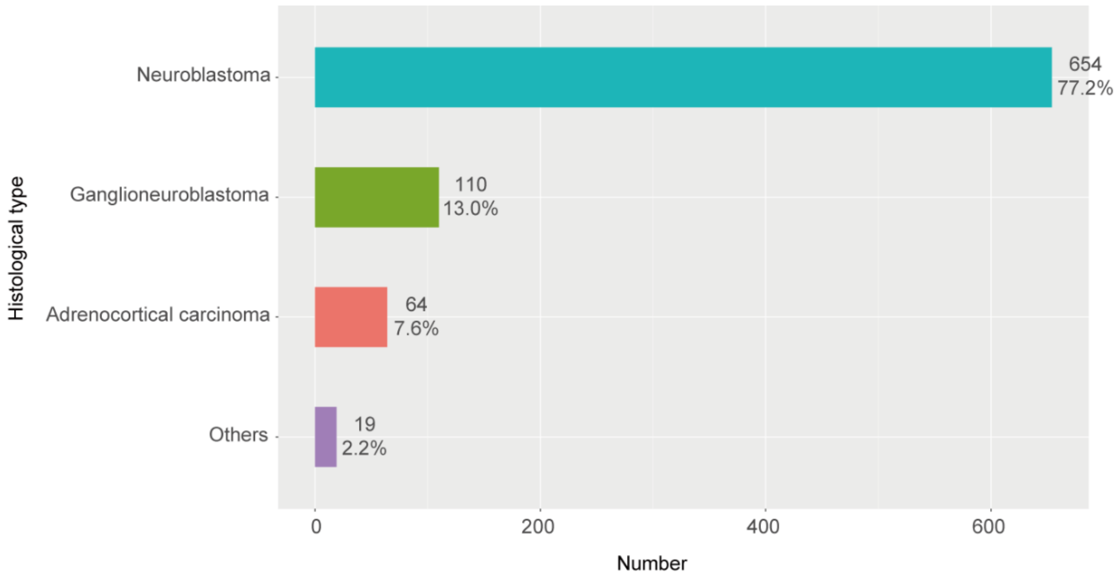

Figure S3. Density plots of age at diagnosis in all enrolled patients.

(A) Distribution in all patients, male and female subgroups. (B) Distribution in different histological types.

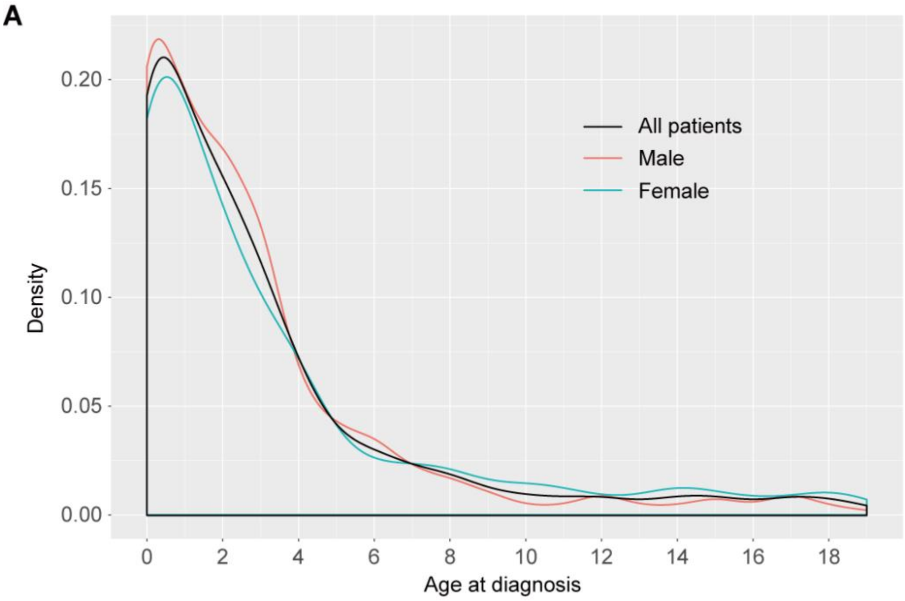

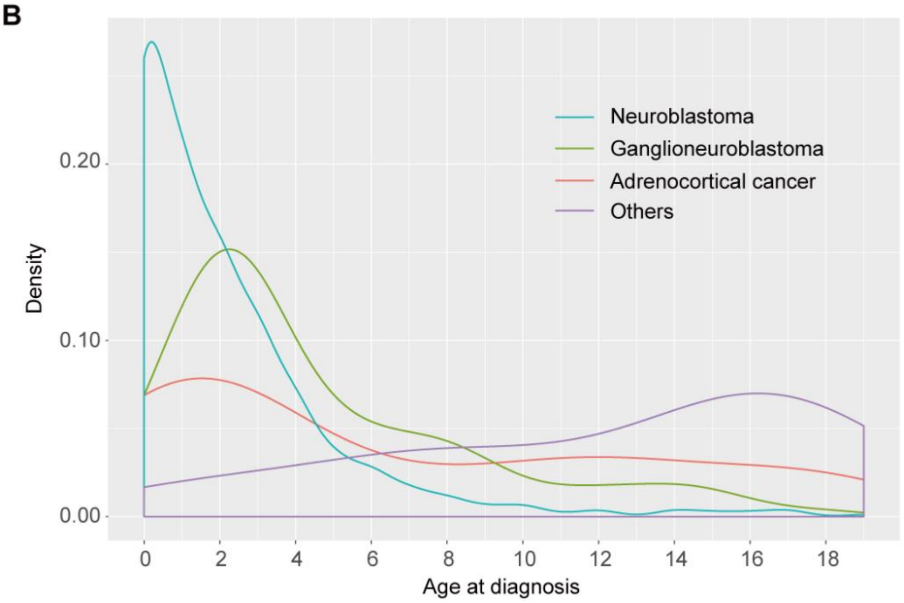

Figure S4. Kaplan-Meier survival curves of all enrolled patients categorized into different histological type groups.

(A) Kaplan-Meier survival curves for OS. (B) Kaplan-Meier survival curves for CSS. ACC: adrenocortical cancer; NB: neuroblastoma; GNB: ganglioneuroblastoma.

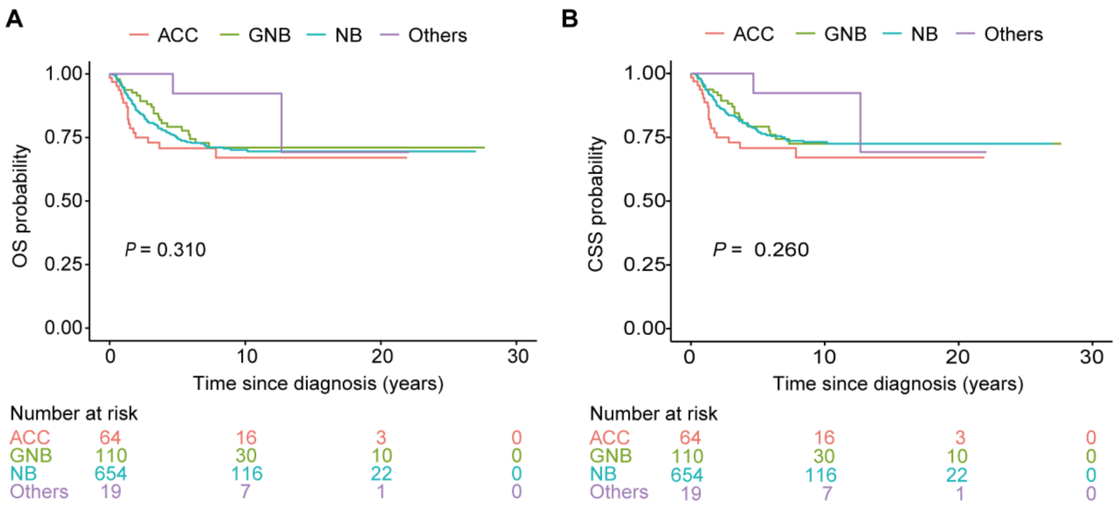

Figure S5. X-tile plots identifying the optimal risk score cutoff based on OS and CSS.

(A, D) X-tile plots for the training set. The coloration of the plot represents the strength of the association at each division, ranging from low (dark, black) to high (bright, red). (B, E) The distributions of the number of patients by risk score. (C, F) Kaplan-Meier plots categorized by the low-risk and high-risk groups according to the optimal risk score cutoff.

(A–C) X-tile plots identifying the optimal OS risk score cutoff. The optimal OS risk score cutoff was determined as 2.41 (χ^2 = 113.4, P <0.0001).

(D–F) X-tile plots identifying the optimal CSS risk score cutoff. The optimal CSS risk score cutoff was determined as 2.48 (χ^2 = 107.7, P <0.0001).

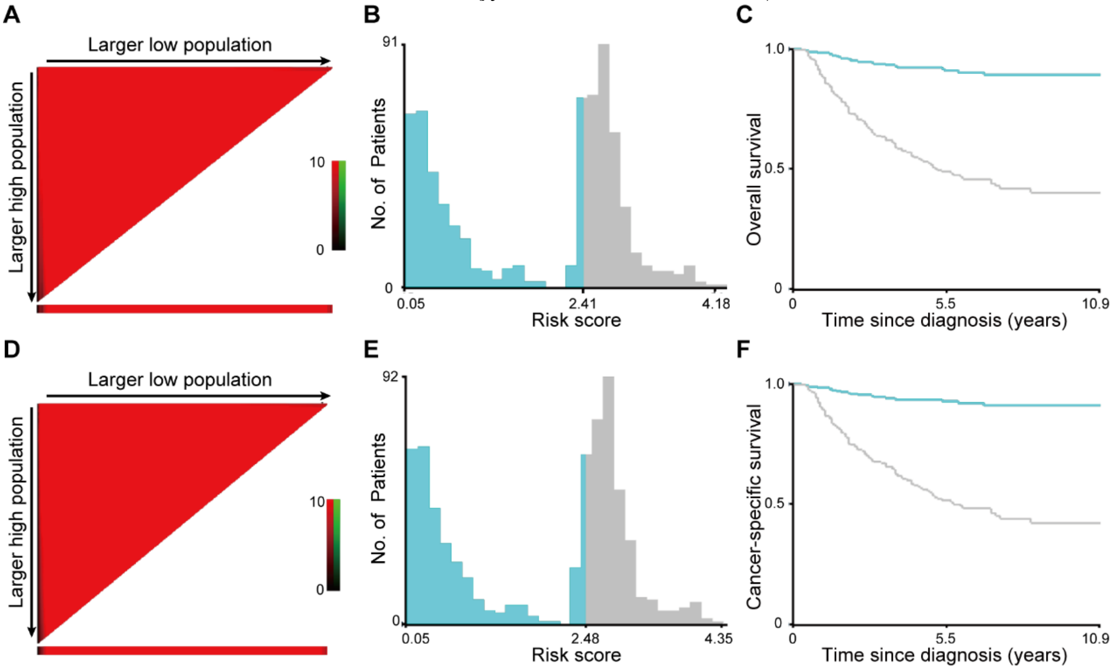

Figure S6. Kaplan-Meier survival curves (OS) categorized into low-risk and high-risk groups in stratified analyses in the combined training and validation set.

Significant discrimination between the OS of the high-risk and low-risk patients was observed in various subgroups, including (A, B) sex, (C, D) tumor laterality, (E–G) tumor invasion, (H, I) N stage and (J–L) histological type.

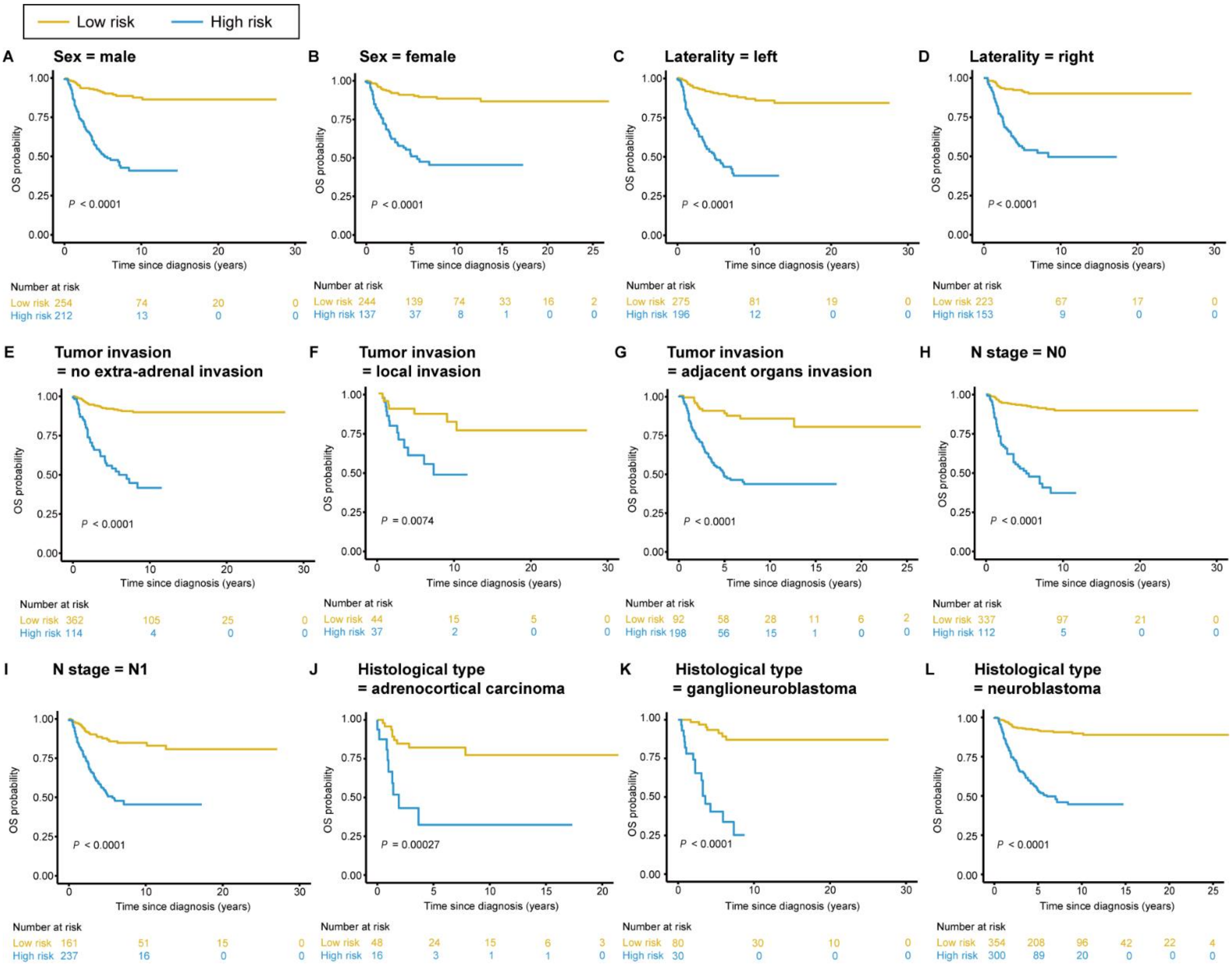

Figure S7. Kaplan-Meier survival curves (CSS) categorized into low-risk and high-risk groups in stratified analyses in the combined training and validation set.

Significant discrimination between the CSS of the high-risk and low-risk patients was observed in various subgroups, including (A, B) sex, (C, D) tumor laterality, (E–G) tumor invasion, (H, I) N stage and (J–L) histological type.

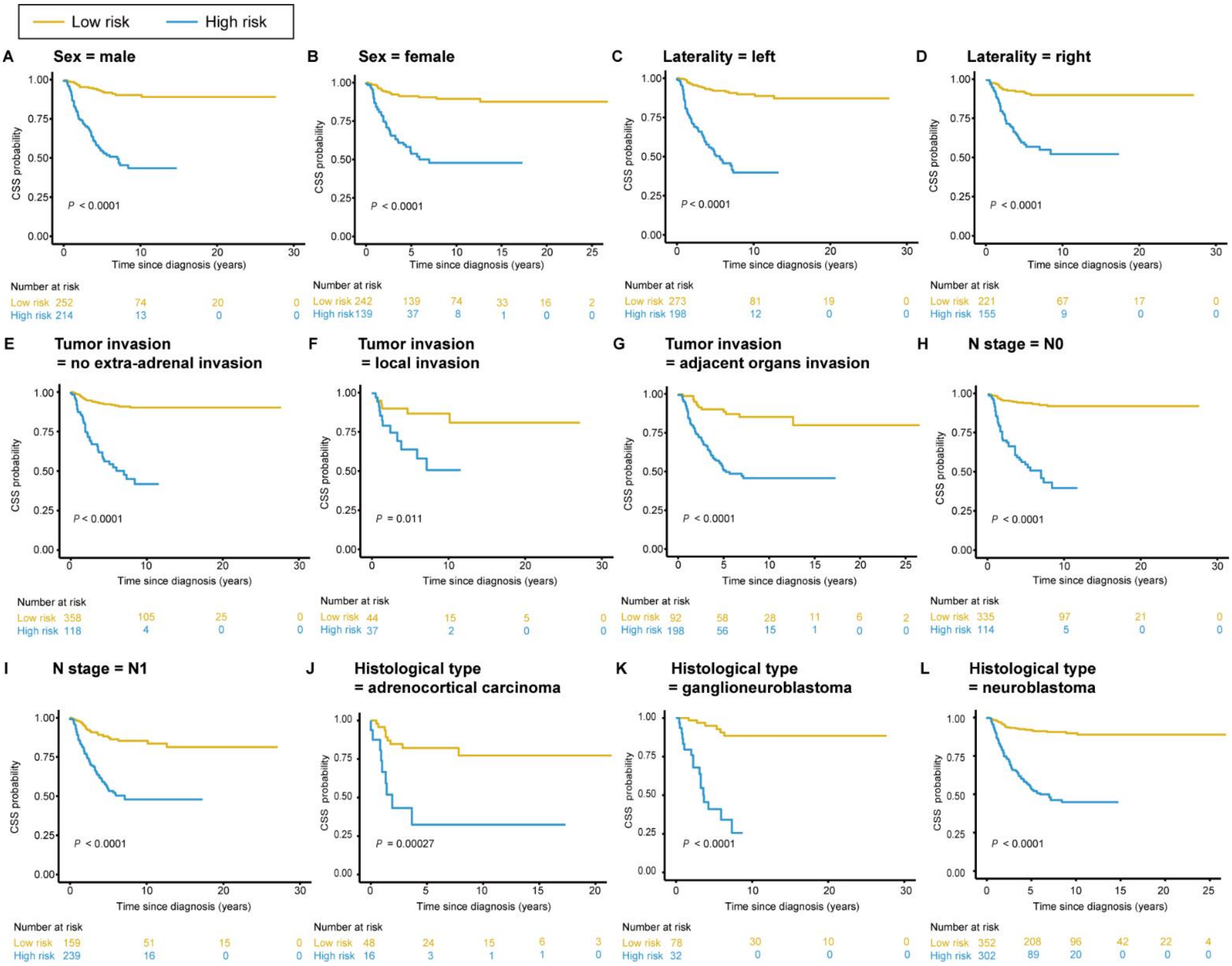

Figure S8. DCA for the nomograms performed in the training and validation sets.

(A, B) DCA for the OS nomogram performed in the training set (A) and in the validation set (B), respectively.

(C, D) DCA for the CSS nomogram performed in the training set (A) and in the validation set (B), respectively.

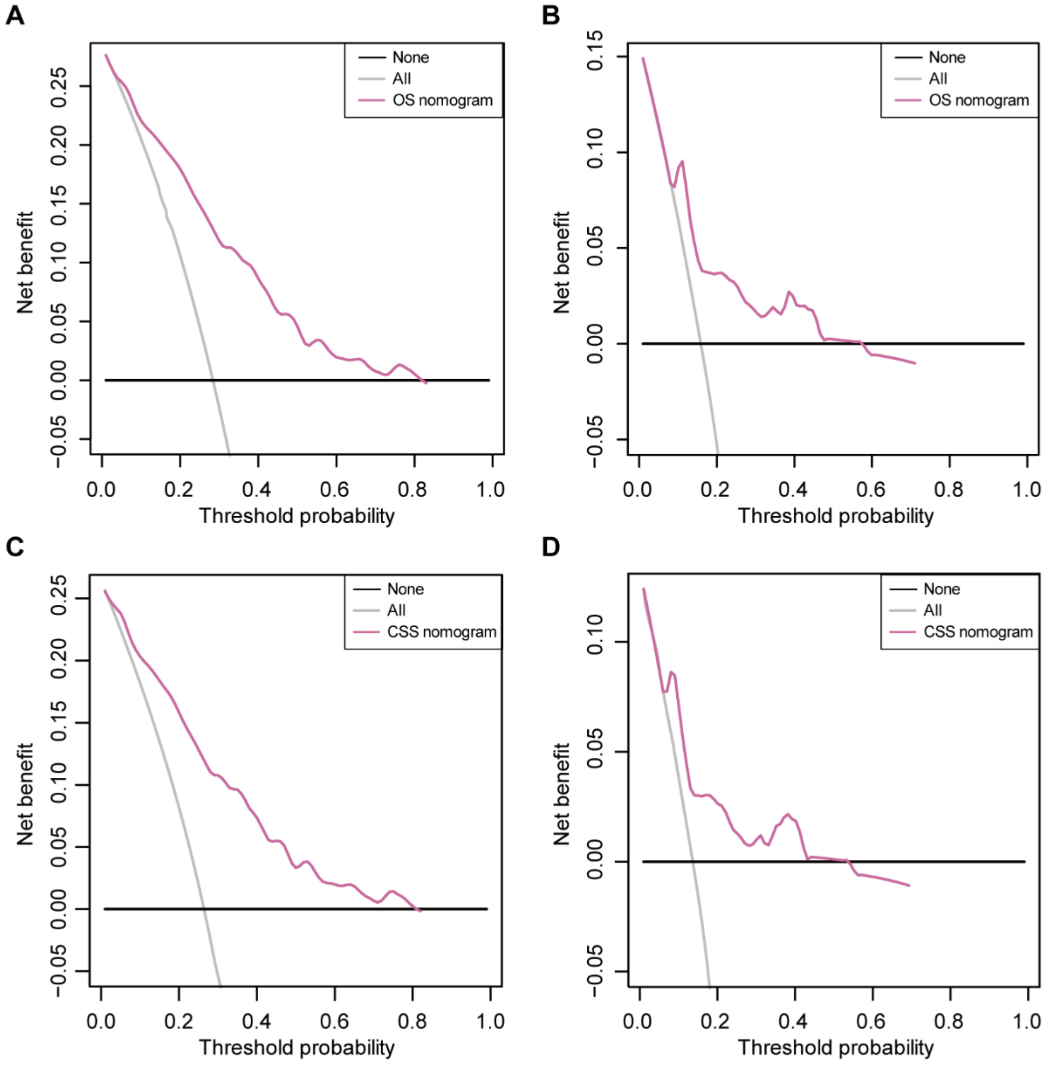

Figure S9. The OS nomogram II and its performance.

(A) The OS nomogram II was developed by additionally incorporating histologic grade. (B) Calibration curves of the OS nomogram II. (C) DCAs comparing the net benefit of the OS nomogram versus the OS nomogram II.

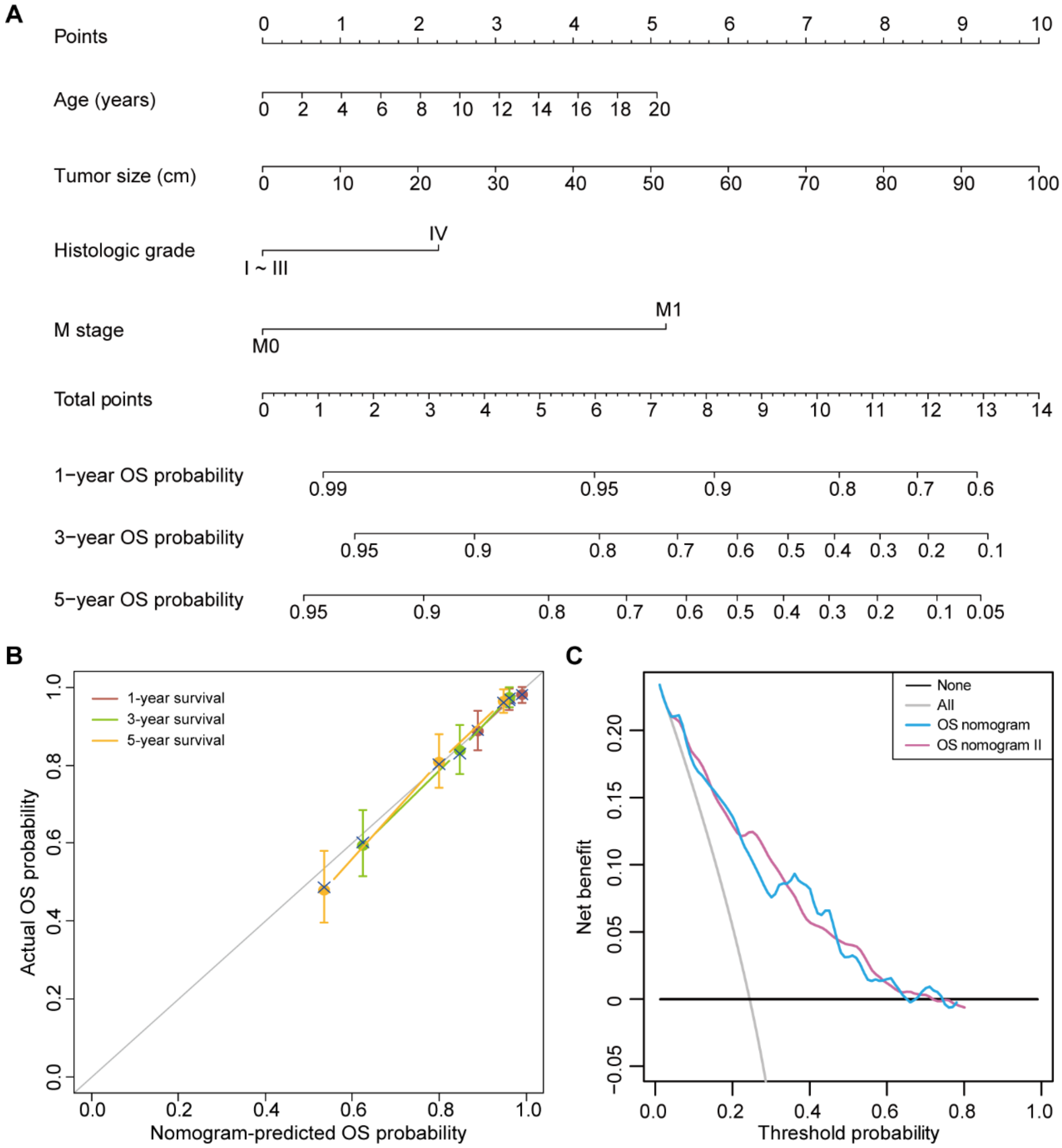

Figure S10. The CSS nomogram II and its performance.

(A) The CSS nomogram II was developed by additionally incorporating histologic grade. (B) Calibration curves of the CSS nomogram II. (C) DCAs comparing the net benefit of the CSS nomogram versus the CSS nomogram II.

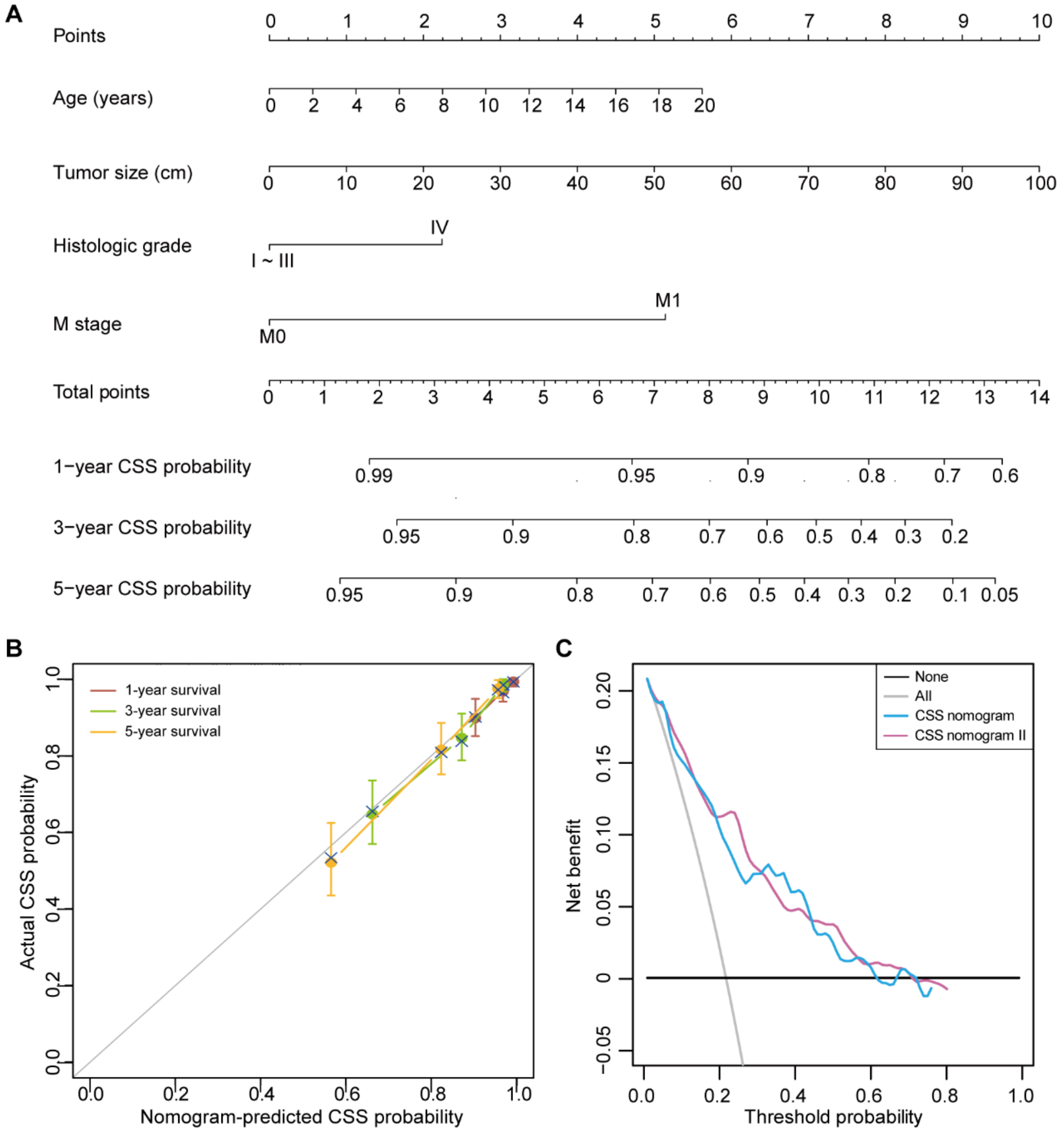

Supplement: Supplementary file 1 — Supplementary figures and table. [file jcav11p2080s1.pdf]
